# Validation study of the apathy motivation index in French adults

**DOI:** 10.3389/fpsyg.2023.1252965

**Published:** 2023-10-20

**Authors:** Xavier Corveleyn, Camille Corbel, Roxane Fabre, Radia Zeghari, Kathy Dujardin, Philippe Robert, Valeria Manera

**Affiliations:** ^1^LAPCOS, Université Côte d'Azur, Nice, France; ^2^MSHS Sud-Est, Maison des Sciences de l'Homme et de la Société Sud-Est, Nice, France; ^3^CoBTeK Lab, Université Côte d'Azur, Nice, France; ^4^Département de Santé Publique, Centre Hospitalier Universitaire de Nice (Public Health Department University Hospital of Nice), Nice, France; ^5^CHU Lille, Lille Neurosciences and Cognition, INSERM, Université Lille, Lille, France; ^6^Centre Hospitalier Universitaire de Nice, Centre mémoire CMRR, Nice, France; ^7^Association Innovation Alzheimer, Nice, France

**Keywords:** apathy, motivation, healthy population, standardization, assessment, self-report scale, prevention

## Abstract

**Objective:**

Apathy is present in many brain disorders, but it is also prevalent to varying degrees in healthy people. While many tools have been developed to assess levels of apathy in pathology, no standardized measure of apathy in healthy people exists.

**Method:**

Therefore, this study aimed to validate the French version of the Apathy Motivation Index (f-AMI). The results of 729 participants were analyzed using an exploratory factorial analysis.

**Results:**

Preliminary analyses suggested that the three domains of apathy extracted in the original AMI scale—behavioral activation (BA), social motivation (SM), and emotional sensitivity (ES)—could be found also in the f-AMI. A further exploratory analysis showed that a higher number of factors could be extracted, particularly for women. Specifically, both social and emotional factors could be divided into two sub-factors: (1) social motivation toward strangers or toward an acquaintance and (2) self-directed emotional sensitivity directed toward others. Regarding construct validity, the scores of f-AMI were correlated with the French Dimensional Apathy Scale results. Concerning the divergent validity, emotional sensitivity in apathy is different from depression, anhedonia, and fatigue levels.

**Conclusion:**

These results suggest that the f-AMI can be used to assess levels of apathy in healthy adults.

## Introduction

In the scientific literature, apathy has traditionally been defined as a diminished level of motivation to act (Marin, 1990, 1996; Robert et al., 2009), as well as an autoactivation deficit (Levy and Dubois, 2006). In line with the diagnostic criteria for apathy in brain disorders updated in 2018 and 2021 (Robert et al., 2018; Miller et al., 2021), apathy is now defined as a clinical syndrome characterized by a reduction in self-initiated goal-directed activity (Chong, 2020). The new diagnostic criteria revised the apathy domains. Specifically, they state that the reduction of goal-directed activity could be observed in behavioral, cognitive, emotional, or social dimensions.

These dimensions may be related to distinct neural substrates, particularly in relation to the prefrontal cortex–basal ganglia networks (Levy and Dubois, 2006; Levy, 2012) and anterior cingulate and ventral striatum disruption (see Le Heron et al., 2018 for review). Deficits in the behavioral and cognitive dimensions consist of changes in different spheres of a patient's life, such as reduced general level of activity, difficulties maintaining an activity or a conversation, or making choices. These patients may be less interested in external issues or in their own personal, family, and friends' wellbeing. Deficits in the emotional dimension consist of a loss or reduction of spontaneous emotions, emotional reactions to the patient's environment, the emotional impact of their own behaviors on others, or empathy. Finally, in the social interaction dimension, researchers and clinicians may observe less spontaneous social initiatives, a reduced response to the stimulated environment in a context of social interaction, less interest in relationships with family members, and less verbal interaction. A reduction in social interactions had been previously mentioned (e.g., Sockeel et al., 2006; Ang et al., 2017), and the social interaction dimension was recognized as a separate dimension in the 2018 criteria.

Apathy was observed in many neuropsychiatric and psychiatric disorders, such as Alzheimer's disease (Zhao et al., 2016), Huntington's disease (Craufurd et al., 2001; Camacho et al., 2018; Andrews et al., 2020), Parkinson's disease (Den Brok et al., 2015; Radakovic et al., 2018), vascular neurocognitive disorders (Staekenborg et al., 2010), amyotrophic lateral sclerosis (Radakovic et al., 2017), stroke and traumatic brain injury (Worthington and Wood, 2018), major depressive disorder (Yuen et al., 2015), schizophrenia (Bortolon et al., 2018), obsessive-compulsive disorder (Raffard et al., 2019), epilepsy (Seo et al., 2017), and human immunodeficiency viruses (Cysique and Brew, 2019). Apathy also shows comorbidities with other clinical syndromes, such as depression, fatigue, and anhedonia (see Zhao et al., 2016; Husain and Roiser, 2018 for reviews). Despite some overlaps between apathy and these syndromes at the clinical, neural, and physiological levels, there is evidence that apathy may represent a separate dimension (Husain and Roiser, 2018).

From a clinical point of view, for most of these diseases, the presence of apathy was associated with a decrease in quality of life with impaired activities of daily living (Yeager and Hyer, 2008; Kamat et al., 2016; D'Iorio et al., 2017; Fritz et al., 2018) and an increase in caregivers' distress or burden (Watermeyer et al., 2015; Kamiya et al., 2017). Finally, apathy was associated with faster cognitive and functional decline in neurodegenerative disorders, for instance, Alzheimer's disease (Starkstein et al., 2006), Huntington's disease (Andrews et al., 2020), Parkinson's disease (Dujardin et al., 2009), or mild cognitive impairment (Papastavrou et al., 2007). For these reasons, identifying apathy is a priority in clinical practice and research.

Apathy was also described to varying degrees in healthy people (Ang et al., 2017; Hezemans et al., 2019), with ~2% of healthy adults classifiable as “generally apathetic,” 12% as “emotionally apathetic” (with sub-clinical symptoms restricted to the emotional dimension), and 25% as “behaviourally/socially apathetic,” [with sub-clinical symptoms restricted to the behavioral or social dimension (Ang et al., 2017)]. While it is hard to determine in non-clinical settings if self-reported apathy represents a clinically relevant symptom (i.e., if the person meets the full spectrum of the diagnostic criteria for apathy, Robert et al., 2018), self-reported apathy in the adult population significantly affects quality of life (Nijsten et al., 2018) and may represent a risk factor for the development of other conditions (for instance, neurodegeneration (Fresnais et al., 2023), suggesting the importance of apathy assessment also in healthy adults. Consequences of apathy in healthy people include, for instance, cognitive deterioration (Kawagoe et al., 2017; Klaasen et al., 2017), a reduced precision of prior beliefs related to action outcomes (Hezemans et al., 2019), a loss of empathy capacities (Lockwood et al., 2017a,b), or a reduction of employment opportunities (Vansteenkiste et al., 2004, 2005; Llinares-Insa et al., 2018). Therefore, self-reports may help detect apathy in many situations (e.g., sub-clinical populations, frailty in elderlies, or job seekers) and allow for a faster reorientation toward a healthcare professional. However, currently, there are no validated assessment instruments of apathy for healthy people available in French. Recently, Ang et al. (2017) developed and validated the Apathy Motivation Index^1^, an 18-item self-report scale specifically designed to detect apathy in healthy people (Ang et al., 2017). The AMI has good psychometric characteristics [internal consistency: α = 0.77; test–retest reliability : α = 0.83; and construct validity with DAS (*r* = 0.62, *p* < 0.01) and AES (*r* = 0.61, *p* < 0.01)]. Interestingly, the AMI includes three subtypes in line with the 2018 diagnostic criteria for apathy (Robert et al., 2018): behavioral activation, social motivation, and emotional sensitivity. The aim of the present study was to validate the French version of the Apathy Motivation Index (f-AMI) in a large sample of healthy French adults. Finally, as in Ang et al. (2017), we consider comorbidity found with apathy as depression, anhedonia, and fatigue.

## Methodology for the french translation of AMI

### Participants and procedure

In the first step, the questions of the AMI were translated into French following the recommended procedure for the cross-cultural validation of psychological questionnaires (Vallerand, 1989). Two reverse translations were performed by independent experts: one from English to French, then one from French to English. A committee composed of six professional experts on apathy assessed this last French to English translation for consistency and refined the French version of the AMI to make it consistent with the meaning sought by Ang et al. (2017). The study protocol was approved by the Ethics Committee (*Comité de Protection des Personnes*—CPP Est III, France, MoTap: RCB ID No. 2017-A01366-4).

We performed a first round of validation on healthy participants. All participants gave electronic informed consent in accordance with the Declaration of Helsinki. The exclusion criteria were self-reported neurological or psychiatric disorders (Ang et al., 2017).

In order to verify the validity of the French version, the questionnaire was implemented on Qualtrics^®^ and posted on several social media sites. A total of 228 participants aged 19 to 80 (151 females, 77 males; mean age = 39 years) completed the French AMI. Several participants reported that some items were potentially ambiguous (i.e., items 3, 13, 17, and 18). Moreover, the psychometric data proved to be insufficient. For instance, the internal consistency was found to be too weak with Cronbach's alpha = 0.57. Furthermore, the psychometric analyses highlighted five factors instead of the expected three. To address these issues, the items considered problematic were modified by the same professional committee involved in the first step. The new questionnaire was completed by 143 new participants aged 18 to 82 (119 women and 24 men; mean age = 34.4 years). Participants reported item 2 as problematic. The internal consistency was again too low (α = 0.69). Furthermore, the psychometric analyses highlighted five factors instead of the expected three. To address these issues, item 2, considered problematic, was modified according to the previously described methodology. The expert committee agreed to standardize this third version of the questionnaire (French Apathy Motivation Index, f-AMI, see Supplementary material 1).

### Methodology for the standardization of the French Apathy Motivation Index

#### Participants

A total of 740 responses were collected. Eleven responses were not kept due to the missing data on age and gender, which were at the end of the questionnaire. The f-AMI was completed by 729 participants (579 women, 150 men, mean age = 30.8 years, age range = 18–68, Table 1). Participants were recruited via social media. All other variables were AMI items, DAS score, QDA score, and MFI score. In order to progress through the questionnaire, respondents had to provide answers, and their choices were restricted to the options provided. Among the 729 typical participants who completed the online questionnaire during the test, 61 participants completed the questionnaire a second time after 2 weeks to assess test–retest reliability.

**Table 1:** Description of the population in the function of DAS, QDA, BDI, Plaisir, and MFI scores and of gender and education.

	**Overall -** ***n** =* **729**				**Female -** ***n** =* **579**		**Male -** ***n** =* **150**			
	**Mean**	**[SD]**	**Min**	**Max**	**Mean**	**[SD]**	**Mean**	**[SD]**	**Student's t**	***p*** **-value**
**Age**	30.8	[10.6]	18	68	30.8	[10.4]	31.0	[11.4]	−0.26	0.788
**DAS score**										
Executive	11.0	[5.1]	0	24	11.1	[5.1]	10.5	[5.2]	1.25	0.211
Emotional	7.2	[4.0]	0	22	6.6	[3.7]	9.4	[4.1]	−8.02	**< 0.001**
Initiative	9.8	[3.8]	0	22	9.7	[3.6]	10.2	[4.3]	−1.45	0.189
Total score	28.0	[9.0]	9	50	27.4	[8.6]	30.1	[10.2]	−0.17	**0.003**
QDA score	1.7	[1.6]	0	5	1.7	[1.6]	1.5	[1.6]	1.7	0.088
BDI score	4.7	[4.4]	0	13	4.8	[4.4]	4.0	[4.1]	2.05	**0.041**
SHAPS score	1.6	[2.2]	0	5	1.6	[2.2]	1.7	[2.1]	−0.57	0.567
**MFI score**										
General / Physical	13.4	[4.6]	4	20	13.9	[4.4]	11.6	[4.7]	5.78	**< 0.001**
Physical fatigue	11.4	[4.6]	4	20	11.7	[4.7]	10.1	[4.2]	3.83	**< 0.001**
Mental fatigue	11.4	[4.6]	4	20	11.6	[4.6]	10.7	[4.7]	1.99	**0.047**
Decrease of activities	10.9	[4.7]	4	20	10.9	[4.7]	10.7	[5.0]	0.47	0.638
Decrease of motivation	10.6	[3.7]	4	20	10.8	[3.7]	9.9	[3.7]	2.64	**0.008**
Total score	56.5	[18.2]	20	100	57.7	[18.0]	51.7	[18.3]	3.61	**< 0.001**
	**n**	**(%)**			**n**	**(%)**	**n**	**(%)**		***p*** **-value**
**Gender**										
Female	579	(79.4)								
Male	150	(20.6)								
**Education**									1.07	0.223
Secondary or less	116	(15.9)			86	(14.9)	30	(20.0)		
Superior	605	(83)			487	(84.1)	118	(78.7)		
Unknown	8	(1.1)			6	(1.0)	2	(1.3)		

Bold *p* values indicate the significance of the results.

#### Material and procedure

The f-AMI scale was completed on the Qualtrics^®^ online platform. Participants were asked to create an anonymous personal identifier. In addition to the f-AMI, participants were also asked to complete a set of established related measures to assess construct validity, in the following order:

- *f-DAS—French dimensional apathy scale* (M'Barek et al., 2020): The f-DAS is a 24-item scale that assesses apathy on three different sub-scales, namely executive, emotional, and behavioral/cognitive initiation. Each item was rated on a 4-point Likert scale, with a higher score indicating greater apathy (0–3: 0 = “Almost Always” and 3 = “Hardly Ever” for positively scored items). The total score for the f-DAS was calculated by summing the responses across all 24 items, and sub-scale scores were obtained by summing the responses within each of its three sub-scales: executive, emotional, and behavioral/cognitive initiation.
- *QDA—Apathy diagnosis questionnaire* (Robert et al., 2018): Five closed questions (yes/no) were extracted from the Diagnostic Criteria for Apathy, as they were found to be the most commonly reported apathy examples in patients with neurocognitive disorders (Manera et al., 2020b; see Supplementary material 2). Individual item scores for the QDA were combined to create a total score.
- *BDI-FS-Fr—Beck's short depression questionnaire* (Alsaleh and Lebreuilly, 2017): The BDI-FS-Fr is a 7-item scale that measures the severity of depression. Each item relates to a symptom of depression and was scored on a 4-point Likert scale (0–3: 0 = least severe and 3 = most severe). A higher total score indicates a higher depression level. The overall score on the BDI-FS-Fr was determined by summing the scores for all seven items.
- *SHAPS—Snaith–Hamilton pleasure scale* (Loas et al., 1997): The SHAPS is a 14-item scale that assesses the ability to experience pleasure. While responses were made on a 4-point scale, for simplicity, Snaith et al. scored each item in a binary manner (0–1: 0 = either “Strongly Agree” or “Agree” and 1 = either “Strongly Disagree” or “Disagree'). A higher score indicates a lower anhedonia level. The total score was computed by adding the binary scores (0 or 1) for all 14 items.
- *MFIS—Modified fatigue impact scale* (Fillion et al., 2003): The MFIS is a 20-item scale that measures how fatigue affects daily life, with each item being rated on a 5-point Likert scale (0–4: 0 = “Never” and 4 = “Almost Always”'). A higher score indicates a lower impact of fatigue on the individual. For the Modified Fatigue Impact Scale (MFIS), we calculated sub-scale scores for each of the five domains by summing the responses within those specific domains, in addition to the overall total score.

Participants completed the first online questionnaire consisting of the above-listed scales. Within a minimum of 10 days and a maximum of 20 days, they were asked to complete a questionnaire (retest), including the f-AMI scale and the apathy diagnostic questionnaire (QDA).

### Statistical analysis

To compare data among male and female participants, Student's *t-*test was used for quantitative variables, and the Khi^2^ test was used for qualitative variables. For the first time, the three sub-scales identified in the Ang article were computed (Ang et al., 2017). Cronbach's alpha was computed for each dimension of the whole population. In the second time, analyses were stratified by gender due to the difference in the factor structure. Principal component analysis (PCA) was first carried out on data from respondents who had answered the whole set of 18 questions. The aim was to identify different independent factors that might explain most of the variance in the sample. The plot of eigenvalues was used to identify the number of dimensions. The “elbow method” was used (Cattell, 1966). After having identified the number of dimensions, an exploratory factorial analysis with an orthogonal rotation (Varimax) was performed to simplify the interpretation of the results obtained on the various factors. The loading values were checked for each dimension. Globally, only items that were substantially loaded (>|0.40|) were selected. Items with a value between |0.30| and |0.40| were tested. Each dimension that emerged from PCA was used to define a sub-scale. The score obtained on each sub-scale was computed by summing up the answers to the items included in the sub-scale (Likert scale from 1 to 4). An overall score was also calculated by summing the sub-scale scores.

The internal consistency method was used to assess the reliability of each sub-scale, and Cronbach's alpha coefficients were computed to establish the internal consistency of each sub-scale. Cronbach's alpha higher than 0.60 was considered good internal consistency (Bullinger, 1996). McDonald's omega index was also indicated (Peters, 2014).

The construct-related validity was determined by assessing the item's convergent validity (the correlation between an item and the dimension to which it belongs had to be >0.40) and its discriminant validity (the correlation between an item and the dimension to which it belongs had to be greater than that obtained with any of the other dimensions).

To evaluate the construct and divergent validity, Spearman's correlation was computed between the scores calculated from the AMI sub-scales and all other measures, such as the DAS score and sub-scores, BDI score, MFI scores and sub-scores, QDA score, and SHAPS score.

For intrinsic validity, we calculated the moderate cutoff for the AMI score and the DAS score. The cutoff corresponds to the mean plus one standard deviation for moderate apathy and the mean plus two standard deviations for severe apathy (Ang et al., 2017). Based on the cutoff, we calculated the sensitivity, specificity, positive predictive value (PPV), and negative predictive value (NPV). After 2 weeks, respondents were asked to respond to AMI items to assess test–retest reliability. Test–retest reliability was established using the test/retest Spearman's correlation, which must be at least 0.60 according to Vallerand (1989).

To evaluate criterion validity, the Kruskal–Wallis test was used to compare the AMI scores to the level of education, and Spearman's correlation was used to test the age of participants.

A *p* < 0.05 was considered significant. All analyses were performed using R i386 3.6.1 software.

## Results

Cronbach's alpha values obtained for the behavioral activation (BA), social motivation (SM), and emotional sensitivity (ES) were 0.80 (male = 0.84 and female = 0.79), 0.70 (male = 0.74 and female = 0.69), and 0.67 (male = 0.68 and female = 0.64), respectively. For each sub-scale, Cronbach's alpha values were lower among female participants than among male participants. Furthermore, among the 18 items of the AMI scale, 9 items were significantly different between male and female participants (Q1, Q3, Q5, Q6, Q7, Q11, Q13, Q16, and Q18, *p* < 0.05) (Table 2). Further analyses were thus stratified by sex.

**Table 2:** Mean score at each item to f-AMI and statistical differences between female and male participants.

		**Overall -** ***n** =* **729**		**Female -** ***n** =* **579**		**Male -** ***n** =* **150**			
	**Items**	**Mean**	**[SD]**	**Mean**	**[SD]**	**Mean**	**[SD]**	**Student's t**	***Q*** **-value**
Q1	Je me sens triste ou bouleversé(e) quand j'apprends des mauvaises nouvelles	1.1	[1.0]	0.9	[0.9]	1.5	[1.1]	−7.073	**< 0.001**
Q2	J'engage facilement la conversation avec n'importe qui	1.8	[1.2]	1.7	[1.2]	1.8	[1.3]	−0.651	0.545
Q3	J'aime bien faire des activités avec des personnes que je connais depuis peu	2.1	[1.2]	2.2	[1.1]	1.9	[1.2]	2.684	**0.016**
Q4	Je propose à mes amis des activités à faire ensemble	1.4	[1.2]	1.4	[1.2]	1.4	[1.2]	0.435	0.664
Q5	Je prends des décisions avec détermination et sans hésitation	2.0	[1.2]	2.0	[1.2]	1.7	[1.2]	2.731	**0.015**
Q6	Après avoir pris une décision. je me demande si j'ai fait le mauvais choix	1.5	[1.2]	1.4	[1.2]	1.8	[1.2]	−2.926	**0.012**
Q7	Au cours des deux dernières semaines. je dirais que je me préoccupe beaucoup de ce que mes proches pensent de moi	1.9	[1.3]	1.8	[1.3]	2.2	[1.3]	−3.470	**0.009**
Q8	Je sors avec des amis toutes les semaines	2.2	[1.4]	2.2	[1.4]	2.0	[1.4]	1.621	0.189
Q9	Quand je décide de faire quelque choses. je m'y mets facilement	1.8	[1.2]	1.8	[1.2]	1.86	[1.2]	−0.771	0.567
Q10	Je n'aime pas paresser	2.2	[1.3]	2.2	[1.3]	2.1	[1.3]	1.244	0.35
Q11	Je fais les tâches quand elles doivent être faites. sans que l'on me le rappelle	1.5	[1.2]	1.5	[1.2]	1.7	[1.2]	−2.330	**0.04**
Q12	Quand je décide de faire quelque chose. je suis motivé(e) à le terminer	1.2	[1.1]	1.1	[1.1]	1.1	[1.1]	0.785	0.6
Q13	Je me sens vraiment mal si je dis quelque chose de blessant	0.9	[1.0]	0.8	[1.0]	1.1	[1.1]	−3.093	**0.012**
Q14	J'initie la conversation spontanément	1.7	[1.2]	1.7	[1.1]	1.8	[1.2]	−0.791	0.644
Q15	Si je dois faire quelque chose. je le fais immédiatement afin que ce soit réglé	1.9	[1.2]	1.9	[1.2]	2.0	[1.2]	−0.734	0.521
Q16	J'ai de la peine lorsque j'apprends qu'une connaissance a eu un accident ou est tombé malade	0.7	[0.9]	0.7	[0.8]	0.9	[1.0]	−2.944	**0.014**
Q17	J'aime avoir le choix parmi plusieurs activités	1.0	[0.9]	1.0	[0.9]	0.9	[0.9]	0.751	0.544
Q18	Si je me rends compte que j'ai été désagréable avec quelqu'un. je me sens terriblement coupable	0.9	[1.0]	0.9	[1.0]	1.1	[1.0]	−2.945	**0.011**

The q-value is a statistical measure used in the context of multiple hypothesis testing. It represents the false discovery rate (FDR) associated with a given p-value or significance level (Benjamini and Hochberg, 1995; Storey, 2002). Bold Q values indicate the significance of the results. The original English version of the apathy motivation Index is available online at: https://doi.org/10.1371/journal.pone.0169938.s003, Ang et al. (2017), CC BY.

### Factorial analysis

For male participants, the obtained results were very close to the initial study (Ang et al., 2017). The difference was about the SM factor, where items 8 and 17 had loading values lower than |0.30|. Cronbach's alpha was 0.80 without items 8 and 17, and 0.74 with items 8 and 17. Due to the good internal consistency with the initial SM factor, we decided to leave the two items in the SM factor. The means obtained for the BA, SM, and SE factors were 1.74 (SD = 0.90), 1.64 (SD = 0.79), and 1.43 (SD = 0.69), respectively (Table 3). The three factors selected explained 41% of the variance. While BA correlated positively with SM (*Rho* = 0.466, *p* < 0.001) and negatively with ES (*Rho* = −0.20, *p* = 0.01), ES did not associate with SM (*p* = *0.9*4).

**Table 3:** Result of exploratory factor analysis for male and female participants.

	**Male -** ***n** =* **150**							**Proposed cutoff**	
	**Cronbach's alpha coefficient**	**McDonald's Omega**	**Score (Mean** +**/– SD)**	**Median value**	**Observed score ranged**	**Flow**, ***n*** **(%)**	**Ceiling**, ***n*** **(%)**	**Moderate (Mean** + **1 SD)**	**Severe (Mean** + **2 SD)**
F1 - BA	0.84	0.85	1.74 (+/– 0.90)	1.7	0–4	4 (2.7)	1 (0.7)	≥ 2.64	≥ 3.54
F2 - SM	0.74	0.75	1.64 (+/– 0.79)	1.7	0–3.7	2 (1.3)	3 (2.0)	≥ 2.43	≥ 3.22
F3 - ES	0.68	0.68	1.43 (+/– 0.69)	1.3	0.17–4	2 (1.3)	2 (1.3)	≥ 2.12	≥ 2.81
Total scale	0.75	0.73	1.60 (+/– 0.51)	1.6	0.44–3.11	1 (0.7)	1 (0.7)	≥ 2.11	≥ 2.62
	**Female -** ***n** =* **579**							**Proposed cutoff**	
F1 - 5 items	0.80	0.80	1.70 (+/**–** 0.87)	1.6	0–4	13 (2.2)	6 (1.0)	≥ 2.57	≥ 3.44
F2 - 4 items	0.69	0.72	0.82 (+/– 0.65)	0.8	0–3.75	62 (10.7)	1 (0.2)	≥ 1.47	≥ 2.12
F3 - 3 items	0.77	0.79	1.87 (+/– 0.94)	1.7	0–4	19 (3.3)	7 (1.2)	≥ 2.81	≥ 3.75
F4 - 3 items	0.65	0.67	1.73 (+/– 0.94)	1.7	0–4	20 (3.5)	7 (1.2)	≥ 2.67	≥ 3.61
F5 - 2 items	0.65	0.66	1.83 (+/– 1.15)	2.0	0–4	48 (8.3)	29 (5.0)	≥ 2.98	≥ 4.13
Total scale	0.66	0.59	1.52 (+/– 0.43)	1.5	0.39–2.89	1 (0.2)	1 (0.2)	≥ 1.95	≥ 2.38

For female participants, the results obtained seemed to be different from the Ang et al. (2017) analysis. Using the eigenvalue screen plot and the elbow method (see Supplementary material 3), five factors emerged. The five factors selected explained 46% of the variance. The first factor contained five items (Q9, Q10, Q11, Q12, and Q15), with Cronbach's alpha equal to 0.80. These items were all in the BA sub-scale (F1). The mean score obtained was 1.70 (SD = 0.87) (Table 3). The second factor (outward emotional sensitivity, F2) was created with four items (Q1, Q3, Q16, and Q18). Initially, they were in the ES sub-scale. Cronbach's alpha was 0.69, and the mean score was 0.82 (SD = 0.65). The third factor (social motivation toward strangers, F3) was created using three items (Q2, Q3, and Q14). Cronbach's alpha was 0.77, and the mean score was 1.87 (SD = 0.94). These items were on the SM sub-scale. The fourth factor (self-directed emotional sensitivity, F4) was calculated using three items (inversed Q5, Q6, and Q7). Cronbach's alpha was 0.65, and the mean score was 1.73 (SD = 0.94). Initially, Q5 was in the BA sub-scale, and Q6 and Q7 were in the ES sub-scale. The fifth and last factor (social motivation toward an acquaintance, F5) was calculated using items Q4 and Q8. Cronbach's alpha was 0.65, and the mean score was 1.83 (SD = 1.15). The two items were in the SM sub-scale.

F1 was positively associated with F3 and F5 (*Rho* = 0.145 and 0.137, *p* < = 0.001), negatively associated with F4 (*Rho* = −0.290; *p* < 0.001) but not correlated with F2 (*p* = *0.5*73). F2 was positively correlated with F4 (*Rho* = 0.252, *p* < 0.001) but not correlated with F3 and F5 (*p* = 0.991 and *p* = 0.193). F3 was negatively correlated with F4 (*Rho* = −0.289, *p* < 0.001) and positively correlated with F5 (*Rho* = 0.336, *p* < 0.001). To finish, F4 was negatively correlated with F5 (*Rho* = −0.121, *p* = 0.004).

In each of the classes of dimensions, all the items met both the convergent validity criterion and the discriminant validity criterion for male and female participants (Tables 4, 5).

**Table 4:** Convergent validity criterion and the discriminant validity criterion for male participants.

	**Male**					
	**Factors**			**Pearson's correlations for convergent and discriminant validity**		
	**1**	**2**	**3**	**Dimension 1**	**Dimension 2**	**Dimension 3**
AMI 1	-	-	**0.445**	−0.04	0.07	**0.71**
AMI 2	0.161	**0.882**	-	0.39	**0.87**	−0.03
AMI 3	0.161	**0.471**	0.196	0.24	**0.70**	0.20
AMI 4	0.344	**0.475**	-	0.45	**0.72**	0.05
AMI 5	**0.601**	0.289	−0.223	**0.74**	0.43	−0.23
AMI 6	**−0.384**	−0.162	**0.338**	**−0.57**	−0.22	0.27
AMI 7	−0.164	-	**0.332**	−0.21	−0.03	**0.63**
AMI 8	-	0.219	-	0.12	0.31	0.05
AMI 9	**0.711**	0.230	-	**0.76**	0.42	−0.09
AMI 10	**0.464**	0.181	-	**0.62**	0.30	−0.05
AMI 11	**0.744**	-	-	**0.73**	0.24	−0.02
AMI 12	**0.660**	0.209	−0.104	**0.75**	0.38	−0.06
AMI 13	-	-	**0.759**	−0.12	0.02	**0.73**
AMI 14	0.247	**0.865**	-	0.46	**0.86**	0.01
AMI 15	**0.801**	0.289	-	**0.83**	0.47	−0.03
AMI 16	0.104	0.118	**0.362**	0.08	0.17	**0.54**
AMI 17	0.206	0.191	0.153	0.23	0.24	0.20
AMI 18	-	-	**0.851**	−0.18	0.03	**0.74**
Eigenvalue	3.19	2.41	1.86			
% variance	17.7	13.4	10.4			

Bold values indicate a strong (negative or positive) correlation between the items of the scales and the factors.

**Table 5:** Convergent validity criterion and the discriminant validity criterion for female participants.

	**Female**									
	**Factors**					**Pearson's correlations for convergent and discriminant validity**				
	**1**	**2**	**3**	**4**	**5**	**Dimension 1**	**Dimension 2**	**Dimension 3**	**Dimension 4**	**Dimension 5**
AMI 1	-	**0.302**	-	0.176	-	−0.01	**0.64**	−0.04	0.21	−0.02
AMI 2	0.109	-	**0.871**	−0.118	0.137	0.18	−0.01	**0.87**	−0.29	0.26
AMI 3	-	0.123	**0.406**	−0.118	0.328	0.07	0.08	**0.73**	−0.17	0.34
AMI 4	0.140	-	0.150	-	**0.691**	0.20	0.04	0.33	−0.21	**0.84**
AMI 5	0.320	−0.106	0.236	**−0.537**	0.190	0.38	0.14	0.34	**−0.77**	0.21
AMI 6	-	0.170	−0.117	**0.769**	-	−0.18	0.21	−0.24	**0.82**	−0.09
AMI 7	-	0.135	-	**0.454**	-	−0.12	0.19	−0.12	**0.72**	0.02
AMI 8	-	-	0.124	-	**0.684**	0.05	0.07	0.28	−0.02	**0.88**
AMI 9	**0.675**	-	0.107	−0.188	0.158	**0.78**	−0.02	0.22	−0.31	0.19
AMI 10	**0.463**	0.148	-	-	-	**0.64**	0.11	0.10	−0.07	0.07
AMI 11	**0.764**	-	-	−0.108	-	**0.79**	−0.02	0.05	−0.25	0.05
AMI 12	**0.648**	-	-	−0.161	0.132	**0.75**	−0.03	0.18	−0.27	0.16
AMI 13	-	**0.775**	-	-	-	0.00	**0.78**	0.06	0.14	0.02
AMI 14	0.100	-	**0.792**	−0.154	0.187	0.17	0.04	**0.87**	−0.29	0.28
AMI 15	**0.771**	-	-	-	-	**0.78**	−0.05	0.08	−0.21	0.07
AMI 16	-	**0.364**	-	-	0.162	0.03	**0.65**	0.10	0.09	0.16
AMI 17	-	-	-	−0.100	0.133	0.06	0.02	0.10	−0.12	0.11
AMI 18	-	**0.876**	-	-	-	−0.02	**0.81**	0.01	0.23	0.04
Eigenvalue	2.43	1.70	1.70	1.28	1.24					
% variance	13.5	9.4	9.4	7.1	6.9					

Bold values indicate a strong (negative or positive) correlation between the items of the scales and the factors/dimensions.

### Construct and divergent validity

#### Men

F1 and F2 were positively correlated with the Executive and Initiative DAS sub-scale, DAS total, BDI, QDA total, SHAPS score, all MFI sub-scales, and MFI total (*p* < 0.001) (Table 6). The second sub-scale was also positively correlated with the Emotional DAS sub-scale. The F3 was only positively correlated with the Emotional DAS sub-scale, and negatively correlated with the Executive sub-scale and BDI (*p* < 0.004).

**Table 6:** Spearman's correlation between each f-AMI factor and DAS, BDI, MFI, QDA, and SHARPS score for male and female participants.

	**Male**				**Female**					
	**F1 BA**	**F2 SM**	**F3 ES**	**Total**	**F1**	**F2**	**F3**	**F4**	**F5**	**Total**
	**Rho Spearman**	**Rho Spearman**	**Rho Spearman**	**Rho Spearman**	**Rho Spearman**	**Rho Spearman**	**Rho Spearman**	**Rho Spearman**	**Rho Spearman**	**Rho Spearman**
**Dimensional apathy scale (DAS)**										
Executive	0.72^**^	0.47^**^	−0.23^*^	0.57^**^	0.61^**^	−0.16^**^	0.26^**^	−0.52^**^	0.16^**^	0.42^**^
Emotional	0.1	0.29^*^	0.51^**^	0.43^**^	0.07	0.54^**^	0.13^*^	0.18^**^	0.11^*^	0.35^**^
Initiative	0.63^**^	0.59^**^	−0.01	0.69^**^	0.42^**^	0.04	0.38^**^	−0.25^**^	0.32^**^	0.54^**^
Total	0.68^**^	0.59^**^	0.06	0.75^**^	0.56^**^	0.16^**^	0.38^**^	−0.34^**^	0.28^**^	0.64^**^
**Beck depression inventory (BDI)**										
Total	0.52^**^	0.37^**^	−0.25^*^	0.39^**^	0.28^**^	−0.13^*^	0.33^**^	−0.50^**^	0.28^**^	0.29^**^
**Modified Fatigue Impact Scale (MFI)**										
General / physical	0.51^**^	0.40^**^	−0.15	0.43^**^	0.31^**^	−0.13^*^	0.27^**^	−0.37^**^	0.23^**^	0.28^**^
Physical fatigue	0.55^**^	0.46^**^	−0.06	0.51^**^	0.38^**^	−0.09^*^	0.30^**^	−0.33^**^	0.27^**^	0.38^**^
Mental fatigue	0.58^**^	0.54^**^	−0.14	0.56^**^	0.49^**^	−0.17^**^	0.22^**^	−0.42^**^	0.18^**^	0.33^**^
Decrease of activities	0.70^**^	0.44^**^	−0.11	0.59^**^	0.54^**^	−0.08^*^	0.35^**^	−0.41^**^	0.28^**^	0.50^**^
Decrease of motivation	0.63^**^	0.55^**^	−0.14	0.61^**^	0.47^**^	−0.10^*^	0.32^**^	−0.44^**^	0.26^**^	0.41^**^
Total	0.73^**^	0.58^**^	−0.15	0.66^**^	0.54^**^	−0.13^*^	0.36^**^	−0.47^**^	0.30^**^	0.48^**^
**QDA score**										
Total	0.26^*^	0.31^**^	−0.1	0.26^*^	0.18^**^	−0.06	0.16^**^	−0.30^**^	0.22^**^	0.18^**^
**SHARPS score**										
Total	0.31^**^	0.45^**^	0.07	0.44^**^	0.23^**^	0.11^*^	0.35^**^	−0.27^**^	0.31^**^	0.41^**^

* p < 0.05;

** p < 0.001.

#### Women

F1, F3, and F5 were positively correlated with the Executive and Initiative DAS sub-scales, DAS total, QDA score, pleasure score, and all MFI scores. On the contrary, F2 and F4 were negatively correlated with the Executive DAS sub-scale, BDI, and MFI sub-scale. F4 was negatively correlated with the Initiative DAS sub-scale, DAS total, QDA score, and SHAPS score, whereas F2 was positively correlated with the DAS total and SHAPS score. F2, F3, F4, and F5 were positively correlated with the Emotional DAS sub-scale (Table 6).

### Intrinsic validity

The moderate cutoff (mean + 2SD) proposed for the AMI total score (2.11 for men and 0.95 for women) and the total DAS score (> = 39) was used.

#### Men

Using 2.11 as a cutoff, the sensitivity, specificity, positive predictive value (PPV), and negative predictive value (NPV) were the following: 64, 95, 78, and 90%.

#### Women

Using 1.95 as a cutoff, the sensitivity, specificity, positive predictive value (PPV), and negative predictive value (NPV) were the following: 65, 90, 40, and 96%.

### Test–retest

A total of 61 participants completed the AMI scale for retest analysis (47 female and 14 male participants). Among male participants, test–retest reliability coefficients for the scale and sub-scales were satisfactory, indicating stable responses across time (*rho*_overall_ = 0.87, *rho*_BA_ = 0.97, *rho*_SM_ = 0.84, *rho*_ES_ = 0.84).

Among female participants, globally test–retest reliability coefficients for the scale and sub-scales were satisfactory, with a lower coefficient for F2 and F4 (*rho*_F1_ = 0.72, *rho*_F2_ = 0.54, *rho*_F3_ = 0.70, *rho*_F4_ = 0.58, *rho*_F5_ = 0.69).

### Criterion validity

#### Men

The participants' age was not associated with BA, SM, ES, or total scores (*p*>0.30). The level of education was associated with BA (secondary or less: mean = 2.1, SD = 0.9; superior: mean = 1.6, SD = 0.9; unknown: mean = 2.7, SD = 0.7; *p* = 0.006), SM (secondary or less: mean = 1.9, SD = 0.8; superior: mean = 1.5, SD = 0.8; unknown: mean = 3.0, SD = 0.2; *p* = 0.007), and total scores (secondary or less: mean = 1.8, SD = 0.5; superior: mean = 1.5, SD = 0.5; unknown: mean = 2.7, SD = 0.6; *p* = 0.013), but not with ES (*p* = 0.283).

#### Women

The participants' age was associated with F3 (*Rho* = −0.14, *p* < 0.001), F4 (*Rho* = 0.18, *p* < 0.001), and F5 (*Rho* = 0.13, *p* = 0.002), but not with F1, F2, or total scores (*p* > 0.20). The level of education was associated with F3 (secondary or less: mean = 2.1, SD = 0.9; superior: mean = 1.8, SD = 0.9; unknown: mean = 2.8, SD = 1.3; *p* = 0.013) and total scores (secondary or less: mean = 1.6, SD = 0.4; superior: mean = 1.5, SD = 0.4; unknown: mean = 2.0, SD = 0.6; *p* = 0.008), but not with F1 (*p* = 0.188), F2 (*p* = 0.698), F4 (*p* = 0.984), and F5 (*p* = 0.104).

## Discussion

The objective of this study was to develop and validate the French version of the Apathy Motivation Index (f-AMI). As in the English version, the results collected on the f-AMI highlighted the presence of three domains of apathy: behavioral activation, emotional sensitivity, and social motivation, supported by good psychometric properties. Therefore, the French version of the AMI could be used by clinicians with an adult French-speaking population.

Some differences were found compared to the original study of Ang et al. (2017). First, we performed the exploratory factor analysis separately for men and women. Indeed, the data revealed differences in the distribution of factors. For men, three factors were found, which correspond to the three factors found by Ang et al. (2017). However, two items out of the six associated with the SM factor (8 and 17) presented lower psychometric values. In women, five factors have been highlighted, comprising the three initial factors (BA, SM, and ES). A first difference was observed in the social motivation factor (items 2, 3, 4, 8, and 14), which highlighted two sub-factors. These items seem to differ if the social motivation is directed toward an unknown person (items 2, 3, and 14: social motivation toward strangers) or a known person as a friend (items 4 and 8: social motivation toward an acquaintance). An explanation could be gender differences in the context of perceived danger, often greater among women facing the unknown (Scott, 2003). Therefore, the clinician or researcher should consider this result when addressing specific populations, such as those with post-traumatic stress disorder. Second, the items of emotional sensitivity factor were divided into two sub-factors (items 1, inversed 5, 6, 7, 13, 16, and 18). As for social factors, these sub-factors could correspond to self-directed emotional sensitivity (items inversed 5, 6, and 7) and emotional sensitivity toward others (items 1, 13, 16, and 18). A similar result was found for DAS validation (Radakovic et al., 2018) and application with a difference between “Social Emotional” and “Individual Emotional” (M'Barek et al., 2020). The differences between men and women regarding empathy and emotional regulation are present from birth and stable throughout life; thus, women seem to demonstrate a higher emotional expressivity (Christov-Moore et al., 2014). Therefore, the clinician or researcher should take this into account when addressing specific populations (psychopathology or affective disorder) or situations, as in anorexia nervosa pathologies (Martinez et al., 2014).

Regarding construct validity, data analyses have also been separated between male and female participants. Data from the male participant showed significant positive correlations between the AMI total score and the DAS total score (*Rho* = 0.75), allowing for correct convergent validity. Especially, the analysis of male data showed that both social motivation and behavioral activation factors were positively correlated with both executive and initiative DAS sub-scales. These results seem in accordance with the executive process of “energization” (Stuss, 2011), that is, the initiation and maintenance of task-relevant responses. The correlation between the behavioral activation factor and the initiation and executive DAS sub-scales confirms the link with the conceptualization of apathy (Ang et al., 2017). This correlation suggests that initiating action is related globally to self-activation aspects and higher-level cognitive processes (Levy and Dubois, 2006). A satisfactory convergent validity was obtained with the female participant when we compared the DAS total score and the AMI total score (*Rho* = 0.64; *p* < 0.001). Especially, behavioral activation factor (F1) is positively correlated with the initiation and executive DAS sub-scales (*Rho* = 0.42 and *Rho* = 0.61). Outward emotional sensitivity (F2) and self-directed emotional sensitivity (F4) are positively correlated with the emotional sub-scale (*Rho* = 0.54 and *Rho* = 0.18). Social motivation toward strangers (F3) and social motivation toward an Acquaintance (F5) were positively correlated with all the DAS sub-scales and the total DAS score (*Rho* = 0.38 and *Rho* = 0.28). This last correlation highlighted that the social motivation sub-scale measures a person's engagement in social interactions (Ang et al., 2017). This seems to confirm the decline often observed in apathy, social engagement, and social interactions (Sockeel et al., 2006).

Concerning the *divergent validity*, the correlations between the AMI and the measures of depression, anhedonia, and fatigue reveal satisfactory construct validity. Regarding the measures of depression, the behavioral activation and social motivation factors show positive correlations. Depression and apathy have partially similar consequences in the behavioral and social spheres. However, the negative correlations between the social-emotional factor and BDI confirm the difference between apathy and depression in the emotional process. In other words, these results highlight how difficult it can be to distinguish apathy from depression due to overlapping symptoms, such as a lack of action initiation or social commitment. However, apathy is often characterized by emotional flattening, while depression indicates an emotional disorder often characterized by extreme emotional fluctuations (Brown and Pluck, 2000). Regarding anhedonia, results show significant positive correlations between the scores of the SHAPS and AMI scales, which highlights a positive relationship in the healthy population between pleasure-feeling capacities and the level of apathy on behavioral activation and social motivation. Indeed, decision-making is not only based on the senses but also on feeling (Gendolla, 2017). If their action is perceived as pleasurable, individuals will engage in it. Otherwise, they quit (Mees and Schmitt, 2008). However, there were no correlations for the emotional sensitivity factor and the SHAPS found for men whereas a negative correlation for the emotional sensitivity toward oneself factor (F4) was found for women. As with depression, apathy seems to differ from anhedonia in terms of emotional sensitivity. Regarding the fatigue level, correlation analyses between the AMI score and each MFI sub-scales score indicate a positive correlation for both men (except no correlation for emotional sensitivity) and women (except negative correlation for emotional sensitivity (F2 and F4) with the AMI score as for the English version (Ang et al., 2017). Apathy and fatigue levels seem to be linked. More fatigue was associated with an increased level of apathy. Fatigue can be a symptom of reduced motivation characterized by a lack of energy to perform actions, but few studies have investigated the relationship between apathy and fatigue (Cochrane et al., 2015; Skorvanek et al., 2015). However, as for depression and anhedonia, the emotional sensitivity score was not correlated with the MFI score for men and negatively correlated with two sub-factors for women. To summarize the results of divergent validity, emotional sensitivity in apathy is different from depression, anhedonia, and fatigue levels.

Regarding temporal fidelity, the AMI has shown high test–retest reliability coefficients for the scale and the sub-scales, indicating stable responses over time for men and women.

Finally, despite possible statistical limitation (e.g., social media only and no attention check) improvement, the AMI shows good psychometric quality for clinical use. Therefore, the f-AMI could be used in many contexts, such as surveys in public health, for students, or for testing wellbeing in the workplace. Focus should be placed on differences between men and women according to the clinical context of the evaluation (empathy and relationship to others) and on emotional sensitivity sub-scales. All these data highlight the complexity of the apathetic syndrome and the need to give it importance in research and therapeutic strategy. Once correctly identified, treatments for apathy are emerging (Mitchell et al., 2011) in pharmacology (Scherer et al., 2018) and with repetitive transcranial magnetic stimulation (Padala et al., 2018), often accompanied by an ecopsychosocial (Zeisel et al., 2016) or non-pharmacological approach (Kales et al., 2015). However, there is an absence of established treatments, with solid evidence for apathy (Feldman et al., 2007; Manera et al., 2020a). Understanding and identifying apathy is therefore crucial to advancing research in order to set up clinical trials with a well-defined population and efficient apathy measures.

## Data availability statement

The original contributions presented in the study are included in the article/Supplementary material, further inquiries can be directed to the corresponding author.

## Ethics statement

The studies involving humans were approved by the Comité de Protection des Personnes–CPP Est III, France, 1 MoTap: RCB ID no. 2017-2 A01366-4. The studies were conducted in accordance with the local legislation and institutional requirements. The participants provided their written informed consent to participate in this study.

## Author contributions

CC, VM, and XC designed study. CC, XC, RF, and VM analyzed data. XC and CC wrote the first draft of the manuscript. RF wrote statistical sections of the manuscript. All authors contributed to manuscript revision, read, and approved the submitted version.

## Footnotes

^1^The apathy motivation Index is available online at: https://doi.org/10.1371/journal.pone.0169938.s003.

## Supplementary material

Supplementary material 1 Translation in French of AMI (f-AMI).

Supplementary material 2 QDA - Apathy Diagnosis Questionnaire based on Robert et al. (2018).

Supplementary material 3 Eigenvalue screenplot for male (left) and female (right). Eigenvalues allowed to identify the numbers of dimensions. The “elbow method” was used.

Supplementary material 4 Descriptive statistics on the age distribution of the sample.
