# Supplementary material for: Validation study of the apathy motivation index in French adults
Source: Front Psychol. 2023 Oct 20;14:1252965. doi: 10.3389/fpsyg.2023.1252965 (PMC10624122; doi:10.3389/fpsyg.2023.1252965)
Supplement: Supplementary material 1 — Translation in French of AMI (f-AMI). [file Data_Sheet_1.PDF]

Items		DANS LES 2 DERNIERES SEMAINE :	Complétement vrai	Plutôt vrai	Ni vrai ni faux	Plutôt Faux	Complétement faux
1	ES	Je me sens triste ou bouleversé(e) quand j'apprends des mauvaises nouvelles					
2	SM	J'engage facilement la conversation avec n'importe qui					
3	SM	J'aime bien faire des activités avec des personnes que je connais depuis peu					
4	SM	Je propose à mes amis des activités à faire ensemble					
5	BA	Je prends des décisions avec détermination et sans hésitation					
6	ES	Après avoir pris une décision, je me demande si j'ai fait le mauvais choix					
7	ES	Au cours des deux dernières semaines, je dirais que je me préoccupe beaucoup de ce que mes proches pensent de moi					
8	SM	Je sors avec des amis toutes les semaines					
9	BA	Quand je décide de faire quelque chose, je m'y mets facilement					
10	BA	Je n'aime pas paresser					
11	BA	Je fais les tâches quand elles doivent être faites, sans que l'on me le rappelle					
12	BA	Quand je décide de faire quelque chose, je suis motivé(e) à le terminer					
13	ES	Je me sens vraiment mal si je dis quelque chose de blessant					
14	SM	J'initie la conversation spontanément					
15	BA	Si je dois faire quelque chose, je le fais immédiatement afin que ça soit réglé					
16	ES	J'ai de la peine lorsque j'apprends qu'une connaissance a eu un accident ou est tombé malade					
17	SM	J'aime avoir le choix parmi plusieurs activités					
18	ES	Si je me rends compte que j'ai été désagréable avec quelqu'un, je me sens terriblement coupable					

Traduction de l'AMI. Version du 4 décembre 2019, Nice, CoBTeK et LAPCOS : Corveleyn, X., Manera, V., Zeghari, R., Dujardin, K., Robert, P.
