# Supplementary material for: Validation study of the apathy motivation index in French adults
Source: Front Psychol. 2023 Oct 20;14:1252965. doi: 10.3389/fpsyg.2023.1252965 (PMC10624122; doi:10.3389/fpsyg.2023.1252965)
Supplement: Supplementary material 2 — QDA - Apathy Diagnosis Questionnaire based on Robert et al. (2018). [file Data_Sheet_2.pdf]

CRITERES DIAGNOSTIC DE L'APATHIE
APATHY DIAGNOSTIC CRITERIA

AUTO QUESTIONNAIRE
SELF ASSESSMENT

Durant ce dernier mois et d'une manière continue :

	Oui	Non
Avez vous présenté une diminution de votre niveau d'activité général		
Avez vous une diminution de vos centres d'intérêt ?		
Avez vous l'impression d'être moins affectueux que d'habitude ?		
Prenez vous moins d'initiatives par exemple pour proposer des activités sociales aux autres?		
Avez vous moins d'intérêt pour votre famille ?		

During this last month and present most of the time:

	Yes	No
Have you reported a decrease in your overall activity level		
Do you have a decrease in your interests?		
Do you feel less affectionate than usual ?		
Do you take fewer initiatives, for example, to offer social activities to others?		
Do you have less interest in your family?		

Reproduced with permission from Dr. Philippe Robert
