# Supplementary figures and images for: Validation study of the apathy motivation index in French adults
Source: Front Psychol. 2023 Oct 20;14:1252965. doi: 10.3389/fpsyg.2023.1252965 (PMC10624122; doi:10.3389/fpsyg.2023.1252965)

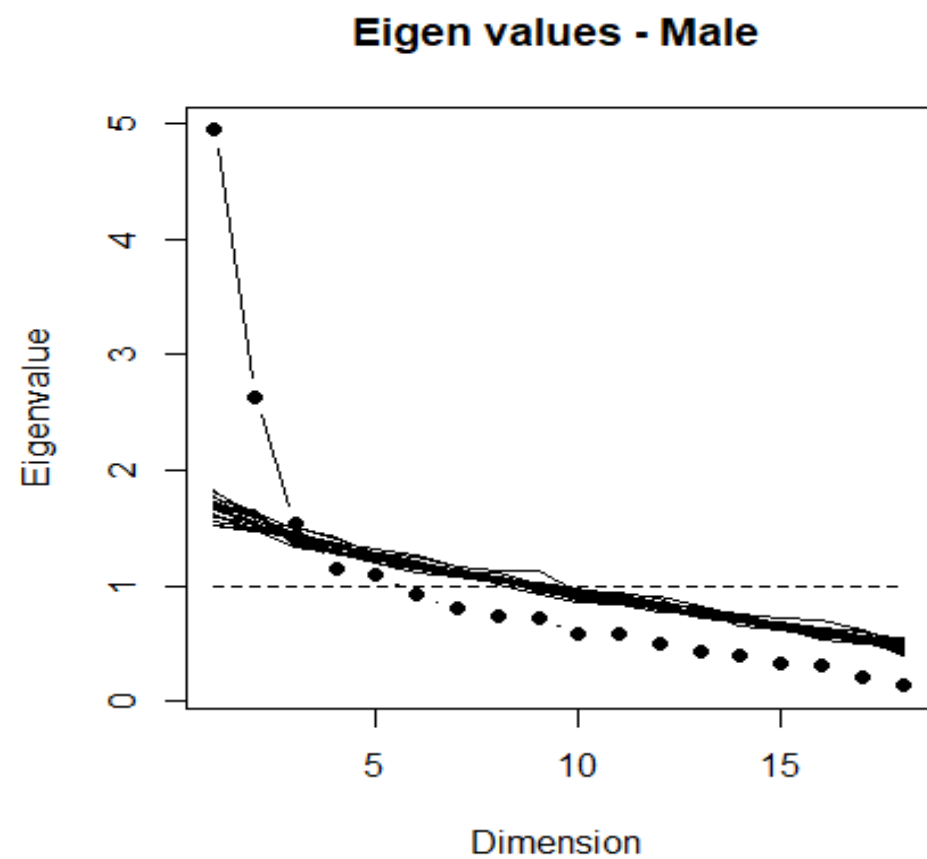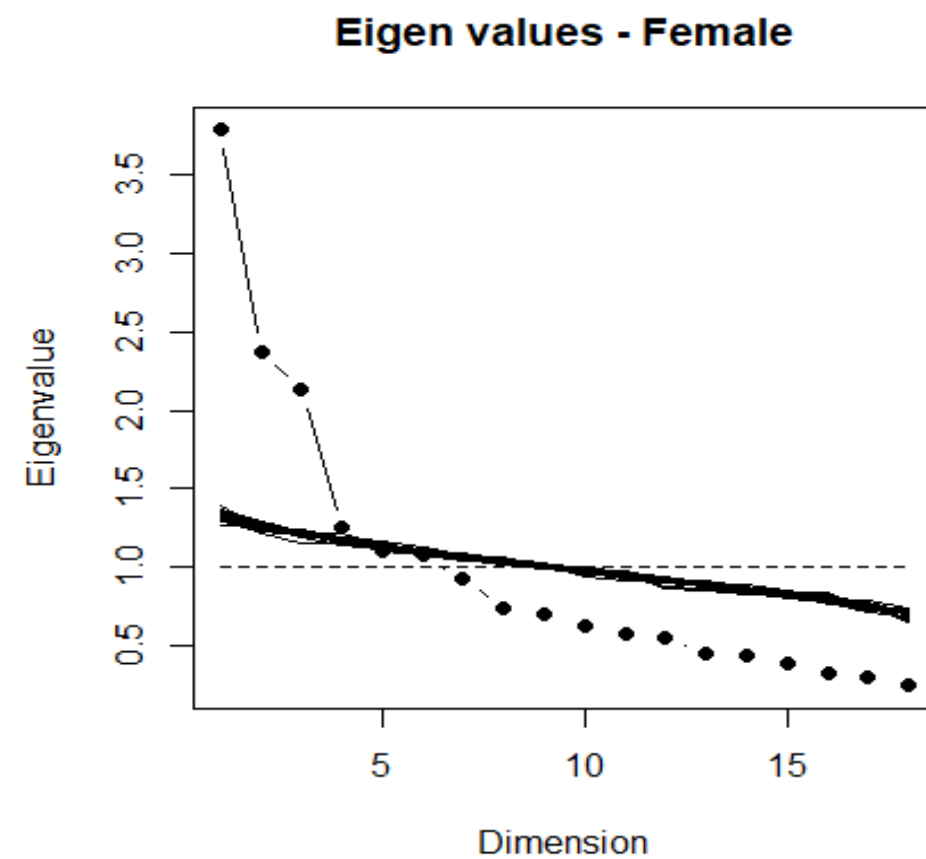

Supplement: Supplementary material 3 — Eigenvalue screenplot for male (left) and female (right). Eigenvalues allowed to identify the numbers of dimensions. The “elbow method” was used. [file Data_Sheet_3.PDF]

## Supplementary material 4. Descriptive statistics on the age distribution of the sample

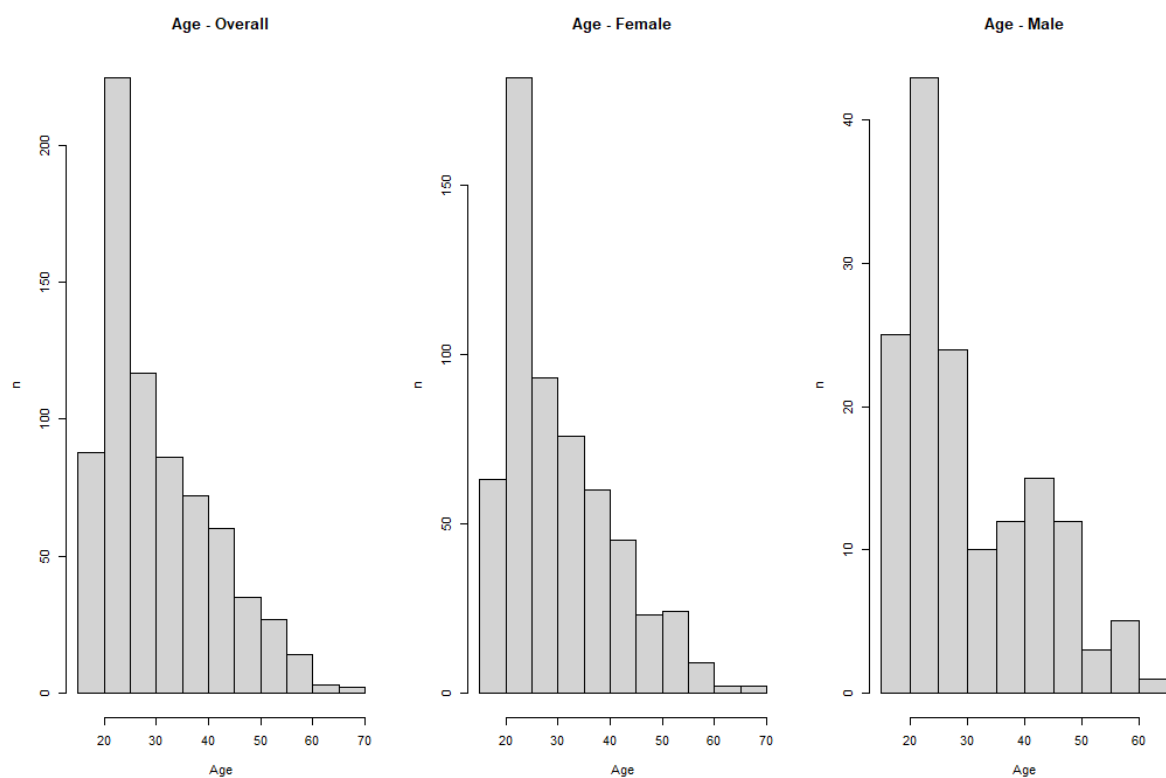

Supplement: Supplementary material 4 — Descriptive statistics on the age distribution of the sample. [file Data_Sheet_4.PDF]
